# Urinary transcription factor 21 (TCF21) as a non-invasive biomarker of podocyte injury in preeclampsia: diagnostic performance and translational insights from a case–control study

**DOI:** 10.1186/s12884-026-09026-z

**Published:** 2026-04-17

**Authors:** Hesham Kamal Habeeb Keryakos, Ayman Moheb Youssuf, Mostafa Abo El-Ela, Nour El-Huda Mohamed Mahdy Abo-Ellil

**Affiliations:** 1 Department of Internal Medicine and Nephrology, Faculty of Medicine, Minia University, Minya, Egypt; 2 Department of Gynecology and Obstetrics, Faculty of Medicine, Minia University, Minya, Egypt; 3 Department of Clinical Pathology, Faculty of Medicine, Minia University, Minya, Egypt

**Keywords:** Preeclampsia, Urinary TCF21, Transcription factor, Podocyte injury, Angiogenic biomarkers, sFlt-1/PlGF ratio, Siagnostic performance, Risk stratification

## Abstract

**Background:**

Early detection of preeclampsia may reduce maternal and fetal morbidity and mortality. While angiogenic biomarkers (sFlt-1 and PlGF) are clinically useful, their sensitivity is imperfect. Podocyte injury is implicated in preeclampsia; urinary transcription factor 21 (TCF21) may serve as a non-invasive marker of glomerular involvement.

**Methods:**

In this case-control study, 160 women with preeclampsia and 64 normotensive pregnant controls matched for age and gestational age were enrolled to evaluate the diagnostic utility of urinary TCF21 and its performance alongside angiogenic markers (sFlt-1, PlGF) at the time of clinical presentation for suspected preeclampsia. Preeclampsia cases were stratified into mild (*n* = 92) and severe (*n* = 68) disease, and each group was further stratified into early-onset and late-onset preeclampsia. Biomarker performance was assessed using logistic regression, receiver operating characteristic (ROC) analysis, and multivariable modeling.

**Results:**

Compared with controls, women with preeclampsia had higher sFlt-1 (11.4-fold; *p* < 0.001), a higher sFlt-1/PlGF ratio (40.6-fold; *p* < 0.001), and higher urinary TCF21 (1.7-fold; *p* < 0.001), with lower PlGF (− 68%; *p* < 0.001). Urinary TCF21 demonstrated moderate discrimination for preeclampsia (AUC 0.769) and was inferior to the sFlt-1/PlGF ratio (AUC 0.812). At the prespecified cutoff of > 370 pg/mL, urinary TCF21 achieved 60.6% sensitivity and 90.6% specificity (LR + 6.45; LR − 0.43). Urinary TCF21 showed an exploratory inverse association with severity (*r* = − 0.258, *p* < 0.001) and diastolic blood pressure (*r* = − 0.244, *p* = 0.002). In multivariable analysis, urinary TCF21 remained independently associated with preeclampsia (adjusted odds ratio 2.83 per 50 pg/mL; 95% CI 1.49–5.59; *p* = 0.002), and combined models improved discrimination (AUC 0.87; 95% CI 0.82–0.91).

**Conclusions:**

Urinary TCF21 is a novel, non-invasive biomarker associated with preeclampsia and showed a hypothesis-generating inverse association with disease severity. In this case-control cohort, its high specificity suggests potential adjunct confirmatory performance at presentation when interpreted alongside angiogenic testing; however, cutoffs and probability thresholds are exploratory and require prospective external validation. Future studies should include creatinine-normalized reporting, assay standardization, and longitudinal sampling to evaluate predictive utility prior to symptom onset.

## Highlights

First clinical evaluation of urinary TCF21 in preeclampsia: This is the first study to investigate urinary transcription factor 21 (TCF21) – a podocyte-specific transcription factor—as a non-invasive biomarker in preeclampsia.

Independent signal beyond angiogenic imbalance: Urinary TCF21 remains significantly associated with preeclampsia after adjustment for established clinical factors and the sFlt-1/PlGF ratio, indicating a distinct renal/podocyte component not captured by angiogenic markers alone.

High specificity suggests adjunct confirmatory potential at presentation: At a cutoff > 370 pg/mL, urinary TCF21 showed 90.6% specificity with a positive likelihood ratio (LR+) of 6.45 in this case-control cohort. These findings are hypothesis-generating and support consideration of urinary TCF21 as an adjunct to angiogenic testing pending prospective external validation.

Novel, hypothesis-generating inverse association with disease severity: Unlike angiogenic biomarkers that typically track severity, urinary TCF21 showed an inverse relationship with preeclampsia severity, which may reflect dynamic biomarker kinetics, podocyte stress responses, altered shedding, or depletion in advanced disease and requires longitudinal validation.

Improved discrimination using a multi-pathway panel: A multivariable model combining urinary TCF21, the sFlt-1/PlGF ratio, and clinical variables demonstrated significantly improved diagnostic discrimination (AUC 0.87, 95% CI 0.82–0.91) compared with models using either pathway alone, thereby integrating signals of placental dysfunction and glomerular injury into a single diagnostic framework.

Translational feasibility for non-invasive stratification: Urine-based detection of TCF21 may offer a practical adjunct to reflect renal/podocyte involvement and refine phenotype characterization at presentation. Clinical implementation will require assay standardization, creatinine-normalized reporting, and validation in independent prospective cohorts.

## Key Message

• Introduces a novel biomarker concept: Establishes urinary TCF21—a podocyte-specific transcription factor—as a new, non-invasive candidate biomarker for preeclampsia.

• Connects placental and renal pathways: Provides clinical evidence that urinary TCF21 captures a renal/podocyte injury signal that is complementary to angiogenic imbalance (sFlt-1/PlGF), supporting a dual-pathway disease model.

• Demonstrates independent diagnostic value: Shows that urinary TCF21 remains independently associated with preeclampsia after adjusting for clinical risk factors and angiogenic markers.

• Identifies a distinctive, hypothesis-generating severity pattern: Urinary TCF21 showed an inverse association with disease severity, suggesting a dynamic podocyte-related signal distinct from the linear severity relationship seen with angiogenic biomarkers; this finding requires mechanistic and longitudinal validation.

• Advances multi-marker risk modeling: Demonstrates improved discrimination when urinary TCF21 is integrated with the sFlt-1/PlGF ratio and clinical variables, supporting a multidimensional panel for diagnosis and stratification.

• Highlights translational practicality: Proposes a feasible urine-based approach for adjunct testing and risk refinement at presentation, with performance and cutoffs requiring assay standardization and external validation before clinical use.

## Introduction

Preeclampsia is a multifactorial disorder unique to pregnancy, complicating up to 5% of pregnancies, and is associated with life-threatening complications in up to 20% of women, especially in preterm preeclampsia (before 37 weeks) [1]. As the second leading cause of maternal mortality after thromboembolic disease, it accounts for approximately 15% of maternal deaths. Current diagnostic criteria rely on new-onset hypertension after 20 weeks’ gestation accompanied by proteinuria (present in about 75% of cases) or end-organ dysfunction, although growing evidence supports proteinuria-independent phenotypes [2, 3].

The pathophysiological model of preeclampsia has evolved from a purely placental-centric “three-stage” model to a systemic disorder. The initial decidual phase features impaired trophoblast invasion and spiral artery remodeling due to deficient pro-angiogenic factors (vascular endothelial growth factor [VEGF], placental growth factor [PlGF]) and reduced heme-oxygenase-1 (HO-1) activity. Subsequent placental hypoxia triggers oxidative stress, characterized by overproduction of anti-angiogenic factors (soluble fms-like tyrosine kinase 1 [sFlt-1], and soluble endoglin [sEng]), pro-inflammatory cytokines, and trophoblast apoptosis. These circulating factors drive generalized maternal endothelial dysfunction, the common final pathway underlying the clinical manifestations of preeclampsia [4].

Maternal cardiovascular adaptation may further modulate disease expression. Normal pregnancy is characterized by vasodilation and plasma volume expansion, leading to a rise in cardiac output (up to ~ 45%) and adaptive left ventricular remodeling. These changes are influenced by placental and fetal signals, including hormones and angiogenic factors such as PlGF and sFlt-1, which regulate vascular tone and facilitate adequate uteroplacental perfusion [5]. In preeclampsia, failure of these compensatory mechanisms can amplify systemic manifestations and end-organ injury.

The kidney is a key target organ in preeclampsia, and podocyte injury appears to contribute to the development of proteinuria. Podocyturia has been reported in women with preeclampsia and may precede overt proteinuria, suggesting that podocyte loss may occur early in the disease course and may be mechanistically linked to glomerular barrier disruption [6]. Transcription factor 21 (TCF21) is a podocyte-enriched regulator involved in glomerular development and maintenance. Experimental and clinical data in proteinuric kidney diseases indicate that TCF21 expression is dynamically regulated during injury: increased TCF21 has been associated with glomerular stress and proteinuria severity, while also participating in protective programs that support podocyte survival and cytoskeletal integrity [7]. These properties make urinary TCF21 a biologically plausible, non-invasive marker of podocyte involvement in preeclampsia, potentially complementing angiogenic biomarkers that primarily reflect placental dysfunction.

Prior studies have evaluated podocyte-related urinary biomarkers in preeclampsia, including nephrin [6], podocalyxin [8, 9], podocyturia [10], and podocyte-specific mRNA markers, but results have been heterogeneous and have not been integrated with angiogenic testing frameworks. TCF21 is a podocyte-enriched transcriptional regulator with a mechanistically distinct role in podocyte stress responses and survival programs, and its urinary signal has not been systematically assessed in preeclampsia. Accordingly, we investigated urinary TCF21 as a complementary renal-axis marker alongside the established placental angiogenic axis (sFlt-1 and PlGF) .

Against this background, we hypothesized that urinary TCF21 could serve as a novel diagnostic adjunct in preeclampsia. The study was designed to assess diagnostic discrimination at presentation rather than early-pregnancy screening or prediction before symptom onset. In this case–control study, we assessed the discriminative performance of urinary TCF21 relative to established angiogenic markers (sFlt-1 and PlGF), and examined its relationship with clinical phenotypes, including disease severity.

## Subjects and methods

This observational case–control study is reported in accordance with the Strengthening the Reporting of Observational Studies in Epidemiology (STROBE) statement.

### Study design, setting, and ethical approval

This observational case–control study examined the association between urinary transcription factor 21 (TCF21) and preeclampsia. The study protocol was reviewed and approved by the Ethics Committee of Minia University (Approval No. 147; 11/2021) and was conducted in accordance with the Declaration of Helsinki. Written informed consent was obtained from all participants prior to enrollment. Participants were recruited from the Obstetrics and Nephrology Departments of Minia University Hospital between November 2021 and February 2023.

### Study participants and grouping

We enrolled 160 pregnant women with preeclampsia (Group I) at 20–37 weeks’ gestation and 64 normotensive pregnant women without hypertension or proteinuria as controls. Controls were matched to cases by age and gestational age to reduce confounding related to gestational timing. Group I was subdivided into:

- Group Ia (mild preeclampsia): *n* = 92 (early-onset < 34 weeks, *n* = 48; late-onset ≥ 34 weeks, *n* = 44).
- Group Ib (severe preeclampsia): *n* = 68 (early-onset < 34 weeks, *n* = 32; late-onset ≥ 34 weeks, *n* = 36).

**Fig. 1:**
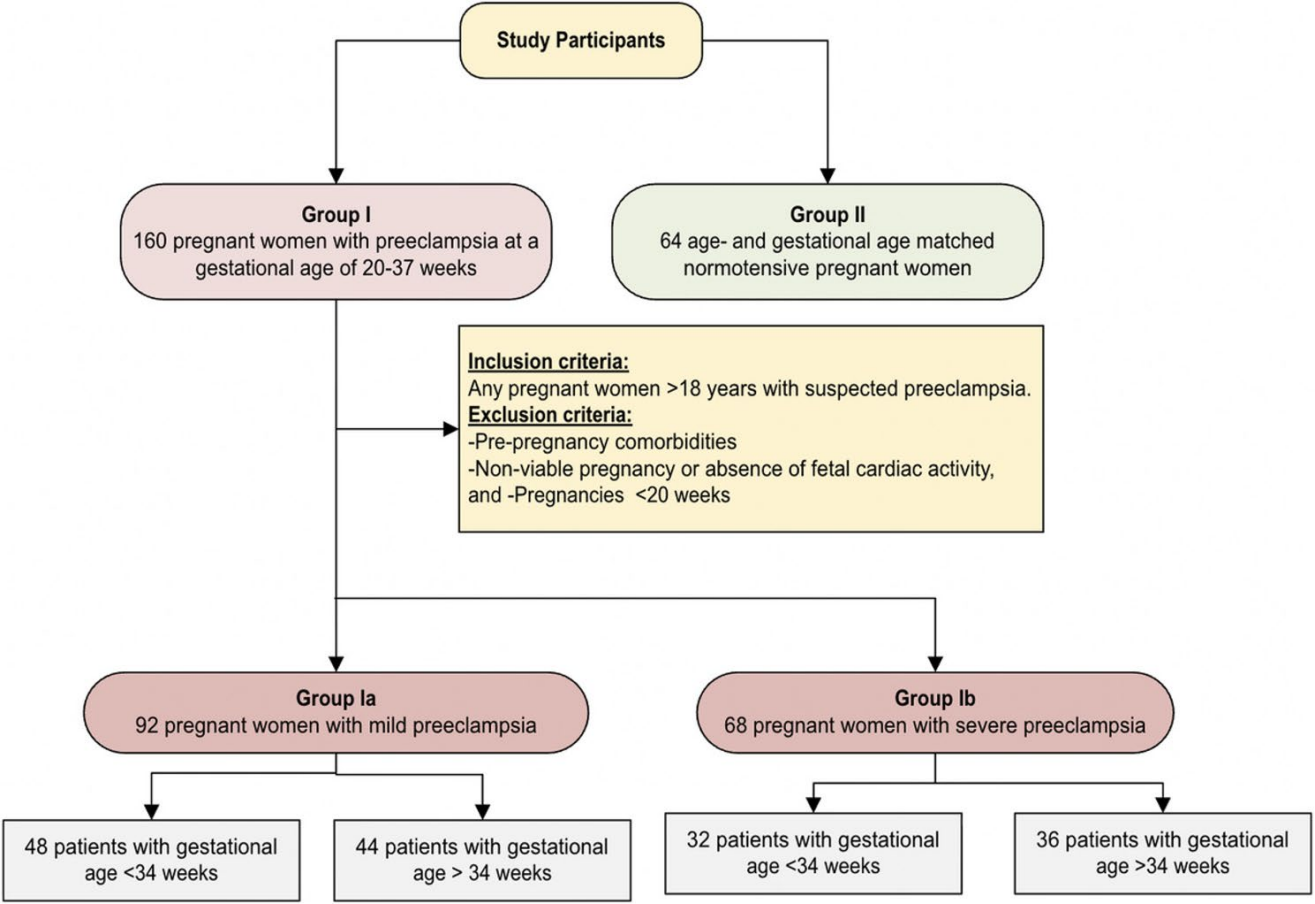
Study flow chart of participant recruitment and grouping. Flow chart showing the total enrolled pregnant women (*n* = 224), divided into preeclampsia cases (*n* = 160) and normotensive controls (*n* = 64). Preeclampsia cases are further stratified into mild (*n* = 92) and severe (*n* = 68) disease, with early-onset (< 34 weeks) and late-onset (≥ 34 weeks) subgroups in each category

The recruitment process and grouping are summarized in Fig. 1.

### Eligibility criteria

Inclusion criteria were pregnant women aged ≥ 18 years with suspected preeclampsia. Exclusion criteria were pre-pregnancy comorbidities, including systemic lupus erythematosus (SLE), diabetes mellitus, preexisting hypertension, chronic heart disease, or chronic kidney disease; non-viable pregnancy or absence of fetal cardiac activity; and gestational age < 20 weeks.

### Diagnostic definitions

Preeclampsia was diagnosed as new-onset hypertension after 20 weeks’ gestation, defined as systolic blood pressure ≥ 140 mmHg and/or diastolic blood pressure ≥ 90 mmHg on two occasions at least 4 h apart in a previously normotensive woman, accompanied by one of the following:

- Proteinuria: ≥300 mg/24-hour urine collection, or urine protein–creatinine ratio (UPCR) ≥ 0.3 g/g, or dipstick ≥ + 1if quantitative methods are unavailable; or.
- In the absence of proteinuria, evidence of maternal end-organ dysfunction, including thrombocytopenia (platelet count < 100,000/mm³), elevated liver transaminases, renal insufficiency (serum creatinine > 1.1 mg/dL or doubling of baseline), pulmonary edema, or new-onset cerebral or visual symptoms.

Severe preeclampsia was defined by any of the following: blood pressure ≥ 160/110 mmHg on two occasions; platelet count < 100,000/mm³; impaired liver function (transaminases ≥ 2× the upper limit of normal and/or severe persistent right upper quadrant/epigastric pain); renal insufficiency (serum creatinine > 1.1 mg/dL or doubling of baseline); pulmonary edema; new-onset cerebral or visual symptoms; eclampsia; or HELLP syndrome (hemolysis, elevated liver enzymes, low platelets) [11].

### Clinical data collection and routine investigations

Demographic characteristics, obstetric history, and maternal–fetal clinical assessments were recorded at enrollment. Lower-limb pitting edema was assessed on physical examination and graded using a standardized pitting scale: absent (0), minimal/trace (1+, Indentation disappears rapidly within 1–2 s), mild (2+, Indentation remains for up to 10–15 s before resolving), moderate (3+; deeper indentation with longer persistence for more than 10–15 s before resolving), and severe (4+; very deep pitting with marked swelling and indentation lasts for more than 30 s). All participants underwent abdominopelvic ultrasonography using a 3.5-MHz transducer (GE Healthcare, USA). Venous blood samples were obtained from all participants at enrollment, and aliquots were stored at − 80 °C until biomarker analysis. Complete blood count (CBC) was measured using an automated hematology analyzer (Sysmex KX-21 N, TAO Medical Inc., Japan). Random blood glucose, liver function tests, and kidney function tests were performed using the Konelab 20i autoanalyzer (Thermo Electron Inc., Finland).

### Urine collection, processing, and storage

At enrollment, participants provided a clean-catch midstream spot urine sample in sterile containers. For preeclampsia cases, sampling was performed at the first clinical evaluation for suspected disease before initiation of specific therapy whenever feasible; for controls, sampling was performed at a routine antenatal visit at matched gestational age. The time of urine collection was recorded, but collection was not restricted to a specific time of day. An aliquot was used immediately for dipstick urine albumin testing and urine protein–creatinine ratio (UPCR) determination according to standard hospital protocols. The remaining urine was centrifuged (3000 × g, 10 min, 4 °C) to remove cellular debris, and the supernatant was aliquoted into low-binding polypropylene tubes and stored at − 80 °C until analysis. All ELISA measurements were performed from a single thawed aliquot; repeat freeze–thaw cycles were avoided. Samples with gross hematuria or evidence of urinary tract infection/contamination on urinalysis (e.g., positive nitrite and/or leukocyte esterase) were excluded from biomarker analysis. This sampling framework was intended to mirror real-world diagnostic workup at presentation rather than pre-symptomatic screening.

### Biomarker measurements

Serum sFlt-1 was measured using a two-site, second-generation enzyme-linked immunosorbent assay (ELISA) kit (*SinoGeneClon Biotech Co. Ltd*,* China*) according to the manufacturer’s instructions. Microtiter plates were coated with monoclonal anti-sFlt-1 antibody. Briefly, 100 µl of standards or samples were added to appropriate wells and incubated for 30 min at 37 °C. The liquid in each well was then removed; 50 µl of an HRP-conjugated polyclonal anti-sFlt-1 antibody (Detection Reagent A) were added to each well and incubated for 30 min at 37 °C. Wells were aspirated and washed with wash buffer, repeating the process five times for a total of five washes, followed by the addition of 50 µl of chromogen solution A and 50 µl of chromogen solution B to each microplate well and incubated for 15 min at 37 °C. The reaction was stopped by adding 50 µL of stop solution to each well, and the color change was measured spectrophotometrically at 450 ± 2 nm. Serial dilutions of recombinant human sFlt-1 were used to establish a standard curve.

Serum PLGF was measured using a similar two-site ELISA kit (*SinoGeneClon Biotech Co. Ltd*,* China*) according to the manufacturer’s instructions. Microtiter plate was coated with monoclonal anti-PlGF antibody. Standards and samples were processed using the same incubation, washing, and detection steps as described above, and a standard curve was generated from recombinant human PlGF. The sFlt-1/ PlGF ratio was calculated to all study participants.

Urinary TCF21 was measured using a two-site second-generation ELISA kit (*SinoGeneClon Biotech Co. Ltd*,* China*) according to the manufacturer’s instructions. Microtiter plates were coated with monoclonal anti-TCF21 antibody. A volume of 100 µl of standards or samples was added to each well and incubated for 30 min at 37 °C. After washing; 50 µl of an HRP-conjugated polyclonal anti-TCF21 antibody (Detection Reagent A) were added to each well and incubated for 30 min at 37 °C. Plates were washed five times, followed by the addition of 50 µl of chromogen solution A and 50 µl of chromogen solution B to each microplate well and incubated for 15 min at 37 °C. The reaction was stopped by adding 50 µL of stop solution to each well, and the color change was measured spectrophotometrically at 450 ± 2 nm. Serial dilutions of recombinant human TCF21 were used to establish a standard curve. Urinary TCF21 values were analyzed as absolute urinary concentrations; creatinine normalization was not performed in this exploratory study.

### Assay quality control and batch management

All biomarker assays were performed in duplicate by laboratory personnel blinded to clinical group. Cases and controls were randomly distributed across plates to minimize batch effects, and each plate included a standard curve and an internal quality-control sample. If duplicate measurements differed by more than 15%, the assay was repeated. Intra-assay and inter-assay coefficients of variation were within the manufacturer-specified limits (Intra-Assay: CV < 8% ‚Inter-Assay: CV < 10% for all biomarkers).

### Statistical analysis

All analyses were performed using IBM SPSS Statistics version 25 (IBM, New York, USA). Continuous variables are presented as mean ± SD if normally distributed or median (IQR) if non-normally distributed. Categorical variables are presented as number (percentage). There were no missing data for variables included in the analyses; therefore, complete-case analysis was performed.

Between-group comparisons used the independent-samples t-test for normally distributed continuous variables and the Mann–Whitney U test for non-normally distributed variables. Categorical variables were compared using the chi-square (χ²) test or Fisher’s exact test, as appropriate. Normality was assessed using the Kolmogorov–Smirnov test.

Associations between continuous variables were assessed using Pearson’s correlation for normally distributed variables and Spearman’s rank correlation for non-parametric variables. Logistic regression was used to evaluate associations between biomarkers and preeclampsia. Biomarkers were scaled a priori for interpretability (sFlt-1 per 100 pg/mL, PlGF per 10 pg/mL, and urinary TCF21 per 50 pg/mL). Because PlGF showed near-complete separation between cases and controls, ridge-penalized logistic regression was additionally applied for models including PlGF, and penalized odds ratios with 95% confidence intervals were reported. Multicollinearity among candidate predictors was assessed using variance inflation factors (VIF), with VIF > 5 considered indicative of problematic collinearity. All tests were two-sided, and *p* < 0.05 was considered statistically significant. Because systolic blood pressure and proteinuria-related metrics contribute to the clinical definition of preeclampsia, models including these variables were interpreted as evaluating incremental value at presentation rather than as stand-alone diagnostic classifiers. We did not perform resampling-based internal validation (e.g., bootstrap or cross-validation); therefore, model performance and thresholds should be interpreted as exploratory pending external validation.

### Sample size and power considerations

An a priori sample size calculation was conducted based on published data for urinary podocyte markers in preeclampsia, which typically report AUC values between 0.70 and 0.80. To detect an AUC of 0.75 (vs. a null AUC of 0.60) with 90% power and a two-sided α = 0.05, approximately 50 cases and 50 controls were required. To ensure sufficient power for subgroup analyses and multivariable modeling, we aimed to enroll at least 150 cases and 60 controls, yielding a case: control ratio of ~ 2.5:1.

During the recruitment period, all eligible women meeting inclusion criteria were invited to participate, resulting in a final cohort of 224 pregnant women (160 preeclampsia cases and 64 controls). Among cases, 81 (50.6%) had early-onset disease (< 34 weeks’ gestation) and 68 (42.5%) had severe preeclampsia, which supported the planned subgroup analyses.

For the primary multivariable logistic regression models predicting preeclampsia, the number of parameters was limited to a maximum of 10, yielding at least 16 events per parameter based on 160 cases, exceeding conventional recommendations (≥ 10 events per parameter) and reducing overfitting risk. Subgroup models (e.g., severe vs. mild) were specified with fewer covariates to maintain acceptable events-per-parameter ratios given 68 severe cases. In a post hoc power assessment using AUC as the effect measure (Hanley–McNeil variance; 160 cases and 64 controls; two-sided α = 0.05), the study had approximately 79% power to detect an AUC of 0.70versus a null AUC of 0.60, 99.5% power for an AUC of 0.75, and > 99.9% power for an AUC of 0.80, confirming adequate power to detect the observed diagnostic performance of urinary TCF21 in this cohort.

## Results

### Demographic, clinical and laboratory characteristics

A total of 224 pregnant women were enrolled, including160 women with preeclampsia and 64 normotensive pregnant controls, who were well matched for age and gestational age (Table 1). Gravidity and parity did not differ significantly between groups, however, women with preeclampsia had a higher number of previous abortions (*p* = 0.007). As expected, preeclampsia was associated with higher systolic and diastolic blood pressures, higher heart rate, and more frequent lower limb edema and convulsions than controls (*p* < 0.001, except convulsions *p* = 0.004).

**Table 1:** Demographics and laboratory characteristics of study participants

		Control *N* = 64	Cases *N* = 160	*p* value
Age (years)	Range	(22–35)	(19–41)	0.837
	Mean ± SD	28.7 ± 4.0	28.5 ± 6.3	
Gravidity	Median	2.5	3.0	0.107
	IQR	(1.0–3.0)	(2.0–4.8)	
Parity	Median	1.5	1.0	0.992
	IQR	(0.0–2.0)	(0.0–3.0)	
Abortion	Median	0.0	0.0	0.007*
	IQR	(0.0–0.0)	(0.0–1.0)	
Gestational age (weeks)	Range	(25–37)	(20–37)	0.981
	Mean ± SD	33.6 ± 3.0	33.1 ± 3.6	
Heart rate (beats/min)	Range	(60–80)	(77–120)	< 0.001*
	Mean ± SD	69.7 ± 5.8	89.5 ± 9.3	
SBP (mm Hg)	Range	(100–120)	(140–210)	< 0.001*
	Mean ± SD	105.9 ± 6.6	155.6 ± 16.4	
DBP (mm Hg)	Range	(60–80)	(80–110)	< 0.001*
	Mean ± SD	66.9 ± 6.4	94.1 ± 6.7	
Lower Limb Edema	No	64 (100%)	0 (0%)	< 0.001*
	Minimal		24 (15.2%)	
	Mild	0 (0%)	65 (41.1%)	
	Moderate	0 (0%)	59 (37.3%)	
	Severe	0 (0%)	10 (6.3%)	
Convulsions	No	64 (100%)	141 (88.1%)	0.004*
	Yes	0 (0%)	19 (11.9%)	
Hb (g/dL)	Range	(10.7–12.8)	(6.6–15.6)	0.222
	Mean ± SD	11.4 ± 0.6	11.1 ± 1.7	
TLC (x10^9^/L)	Median	8.7	10.6	< 0.001*
	IQR	(7.3–9.4)	(8.1–13)	
Platelets (x10^9^/L)	Median	252	188	< 0.001*
	IQR	(229–330)	(140–260)	
Random blood glucose (mg/dL)	Range	(68–100)	(63–133)	< 0.001*
	Mean ± SD	86.1 ± 9.4	100.3 ± 17.1	
INR	Range	(1.0–1.1)	(1.0–2.4)	< 0.001*
	Mean ± SD	1.03 ± 0.04	1.133 ± 0.246	
Serum creatinine (mg/dL)	Median	0.6	0.9	< 0.001*
	IQR	(0.4–0.6)	(0.7–1.0)	
Blood urea (mg/dL)	Median	22.0	29.5	< 0.001*
	IQR	(20.0–23.0)	(23.0–37.8)	
ALT (U/L)	Median	12.0	19.0	< 0.001*
	IQR	(11.0–13.0)	(15.0–32.3)	
AST (U/L)	Median	15.0	22.5	< 0.001*
	IQR	(14.0–16.0)	(16.0–35.0)	
Dipstick urine albumin	Median	0	3	< 0.001*
	IQR	(0–0)	(2–3)	
UPCR g/g	Median	0.08	1.2	< 0.001*
	IQR	(0.07–0.10)	(0.6–2.1)	
sFlt-1 (pg/mL)	Range	222–438	2035–5400	< 0.001*
	Mean ± SD	373.4 ± 62.7	4264.2 ± 804.5	
Serum PlGF (pg/mL)	Range	(160–482)	(11–100)	< 0.001*
	Mean ± SD	236.1 ± 108.6	74.5 ± 22	
sFlt-1/PlGF ratio	Range	(0.8–2.5)	(25.1–481.4)	< 0.001*
	Mean ± SD	1.8 ± 0.6	73.1 ± 66.5	
Urine TCF21 (pg/mL)	Range	(190–400)	125–1225	< 0.001*
	Mean ± SD	300 ± 66.8	512.3 ± 281.4	

Data are presented as mean ± SD (range) or median (IQR) for continuous variables and n (%) for categorical variables

The sFlt-1/PlGF ratio is unitless. p values were calculated using independent-samples t test, Mann–Whitney U test, or chi-square/Fisher’s exact test, as appropriate

*SBP* Systolic blood pressure, *DBP* Diastolic blood pressure, *Hb* Hemoglobin, *TLC* Total leukocytic count, *INR* International normalized ratio, *ALT* Alanine aminotransferase, *AST* Aspartate aminotransferase, *UPCR* Urine protein-creatinine ratio, *sFlt-1* soluble fms-like tyrosine kinase-1, *PlGF* Placental growth factor, *TCF21* Transcription factor 21

* Significant at *p* value < 0.05

Laboratory parameters differed substantially between groups (Table 1). Compared with controls, women with preeclampsia had higher total leukocyte count, lower platelet count, higher INR, higher serum creatinine and blood urea, higher ALT/AST, higher random blood glucose, and markedly higher proteinuria (dipstick and UPCR) (all *p* < 0.001). Hemoglobin did not differ significantly between groups.

**Fig. 2:**
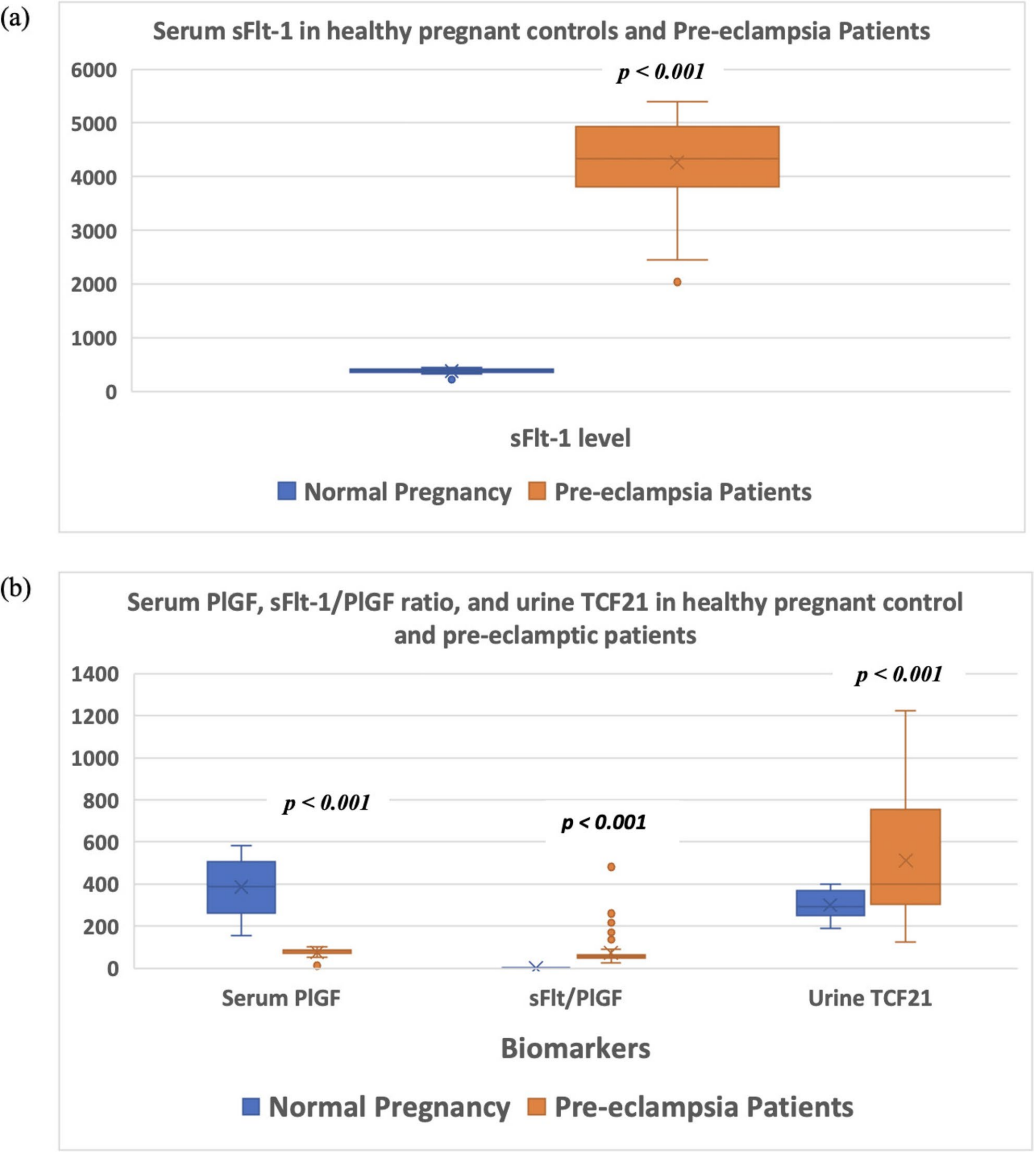
Biomarkers in study participants. (**a**) Mean serum sFlt-1 levels in the study groups. (**b**) Mean serum PlGF, sFlt-1/PlGF ratio, and urinary TCF21 in the study groups

All biomarkers differed significantly between preeclampsia and controls (Table 1; Fig. 2). Preeclampsia was characterized by higher serum sFlt-1, higher sFlt-1/PlGF ratio, higher urinary TCF21, and lower serum PlGF (all *p* < 0.001). Relative to controls, sFlt-1 was 11.4-fold higher, the sFlt-1/PlGF ratio was 40.6-fold higher, urinary TCF21 was 1.7-fold higher, and PlGF was reduced by 68%.

### Correlation of biomarkers in women with preeclampsia with demographic, clinical, and laboratory parameters

Within preeclampsia cases, urinary TCF21 showed inverse associations with diastolic blood pressure (*r* = − 0.244, *p* = 0.002), convulsions (*r* = − 0.193, *p* = 0.014), and preeclampsia severity (*r* = − 0.258, *p* < 0.001), and a weak positive association with platelet count (*r* = 0.194, *p* = 0.014) (Table 2). In contrast, sFlt-1 correlated inversely with age and platelets and was positively associated with severity (Table 2). PlGFshowed an association with convulsions (*r* = 0.162, *p* = 0.041). The sFlt-1/PlGF ratio was positively associated with leukocyte count and severity and inversely associated with gestational age (Table 2).

**Table 2:** Correlation between preeclampsia biomarkers and clinical/laboratory parameters in women with preeclampsia

Cases group	Urine TCF21		Serum sFlt-1		Serum PlGF		sFlt-1/PlGF ratio	
	*r*	*p* value	*r*	*p* value	*r*	*p* value	*r*	*p* value
sFlt-1	-0.021	0.789	--	--	--	--	--	--
PlGF	-0.009	0.912	0.084	0.290	--	--	--	--
sFlt-1/PlGF	-0.057	0.476	0.557	< 0.001*	-0.682	< 0.001*	--	--
Age (years)	0.102	0.198	-0.184	0.020*	0.052	0.510	-0.069	0.387
Gravidity	0.072	0.365	-0.091	0.251	0.037	0.640	-0.069	0.386
Parity	0.107	0.178	-0.098	0.216	-0.017	0.831	-0.050	0.532
Abortion	0.013	0.875	-0.016	0.839	0.102	0.201	-0.064	0.419
Heart rate (bpm)	0.024	0.765	-0.095	0.232	-0.004	0.964	-0.088	0.269
SBP (mm Hg)	-0.151	0.057	-0.029	0.719	-0.020	0.802	0.049	0.542
DBP (mm Hg)	-0.244	0.002*	0.036	0.648	-0.056	0.484	0.105	0.186
Gestational age	-0.092	0.247	0.003	0.971	0.169	0.032	-0.181	0.022*
Hb	-0.036	0.648	-0.083	0.298	0.045	0.573	-0.049	0.539
TLC	-0.125	0.115	0.099	0.211	0.043	0.590	0.158	0.046*
Platelets	0.194	0.014*	-0.231	0.003*	0.058	0.466	-0.098	0.219
Serum creatinine	0.029	0.712	-0.044	0.582	-0.035	0.665	-0.026	0.742
Blood Urea	0.093	0.244	-0.059	0.455	-0.040	0.618	-0.035	0.662
ALT	-0.027	0.739	0.029	0.715	0.036	0.647	-0.035	0.660
AST	-0.050	0.528	0.018	0.823	0.032	0.687	-0.040	0.614
INR	0.090	0.257	0.057	0.470	-0.008	0.918	-0.002	0.977
RBG	-0.091	0.254	-0.044	0.580	-0.114	0.150	-0.016	0.839
Spot UPCR	-0.016	0.845	0.116	0.145	-0.053	0.507	0.081	0.308
Lower Limb Edema	-0.038	0.632	-0.065	0.420	-0.096	0.230	0.001	0.999
Fits	-0.193	0.014*	0.027	0.731	0.162	0.041*	-0.072	0.367
Time of Onset	-0.002	0.977	-0.048	0.551	-0.032	0.692	-0.015	0.853
Preeclampsia Severity	-0.258	< 0.001*	0.163	0.039*	-0.138	0.083	0.181	0.022*

Data are presented as correlation coefficients (r) and corresponding *p* values. Pearson’s correlation was used for normally distributed variables and Spearman’s rank correlation for non-normally distributed or ordinal variables. Ordinal variables (lower limb edema, fits, time of onset, and preeclampsia severity) were coded according to increasing clinical severity

*SBP* Systolic blood pressure, *DBP* Diastolic blood pressure, *Hb* Hemoglobin, *TLC* Total leukocytic count, *INR* International normalized ratio, *RBG* Random blood glucose, *UPCR* Urine protein–creatinine ratio, *sFlt-1* soluble fms-like tyrosine kinase-1, *PlGF* Placental growth factor, *TCF21* Transcription factor 21

*Significant at *p* < 0.05

### Stratification by onset and severity

#### Biomarkers could not differentiate between early- and late-onset preeclampsia

**Fig. 3:**
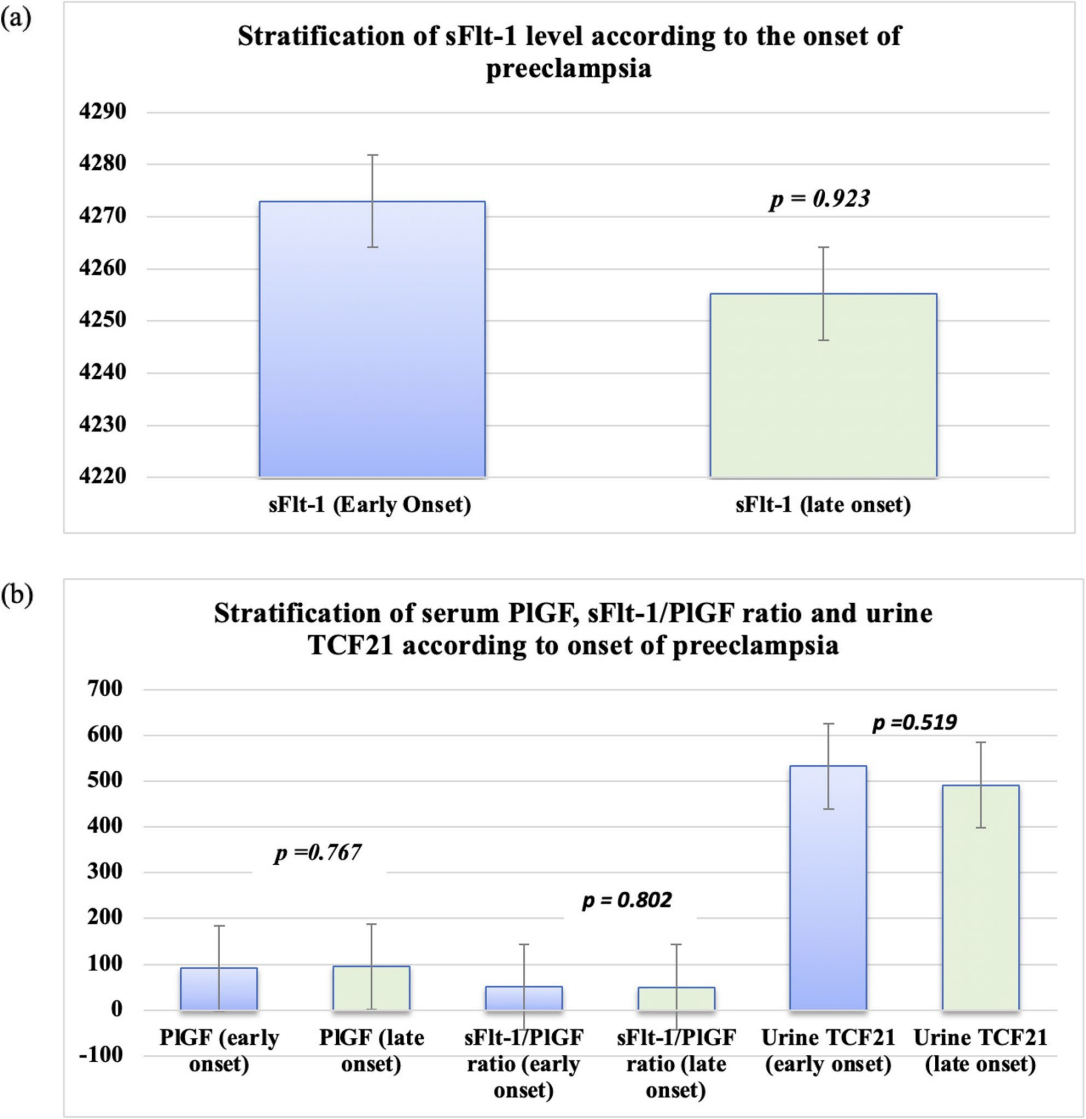
Stratification according to onset of preeclampsia. (**a**) Serum sFlt-1 by onset. (**b**) Serum PlGF, sFlt-1/PlGF ratio, and urinary TCF21 by onset

When cases were stratified by onset, early-onset (< 34 weeks; *n* = 81) and late-onset (≥ 34 weeks; *n* = 79) preeclampsia showed no significant differences in sFlt-1, PlGF, sFlt-1/PlGF ratio, or urinary TCF21 (Table 3; Fig. 3). This supports the interpretation that the observed case–control biomarker signal was not solely driven by gestational timing within the enrolled diagnostic window.

**Table 3:** Levels of angiogenic factors (serum sFlt-1, serum PlGF, sFlt-1/PlGF ratio), and urinary TCF21 levels in early- and late-onset preeclampsia cases

		Early-onset *N* = 81	Late-onset *N* = 79	*p* value
Serum sFlt-1	Range	(2035–5400)	(2590–5400)	0.923
	Mean ± SD	4273 ± 839.36	4255 ± 778.1	
Serum PlGF	Range	55–270	50–250	0.767
	Mean ± SD	91.5 ± 36.47	95 ± 44.27	
sFlt-1/PlGF	Range	16.96–89.55	15.09–78.8	0.802
	Mean ± SD	50.57 ± 15.74	49.71 ± 14.7	
Urine TCF21	Range	125–1225	210–1090	0.519
	Mean ± SD	532.63 ± 312.33	491.41 ± 248.1	

Data are presented as mean ± SD (range). Comparisons between early- and late-onset preeclampsia groups were performed using the independent-samples t test

*sFlt-1* soluble fms-like tyrosine kinase-1, *PlGF* Placental growth factor, *TCF21* Transcription factor 21, sFlt-1/PlGF ratio is unitless

*Significant level at *p* < 0.05

#### Biomarkers may help to stratify the severity of preeclampsia

**Fig. 4:**
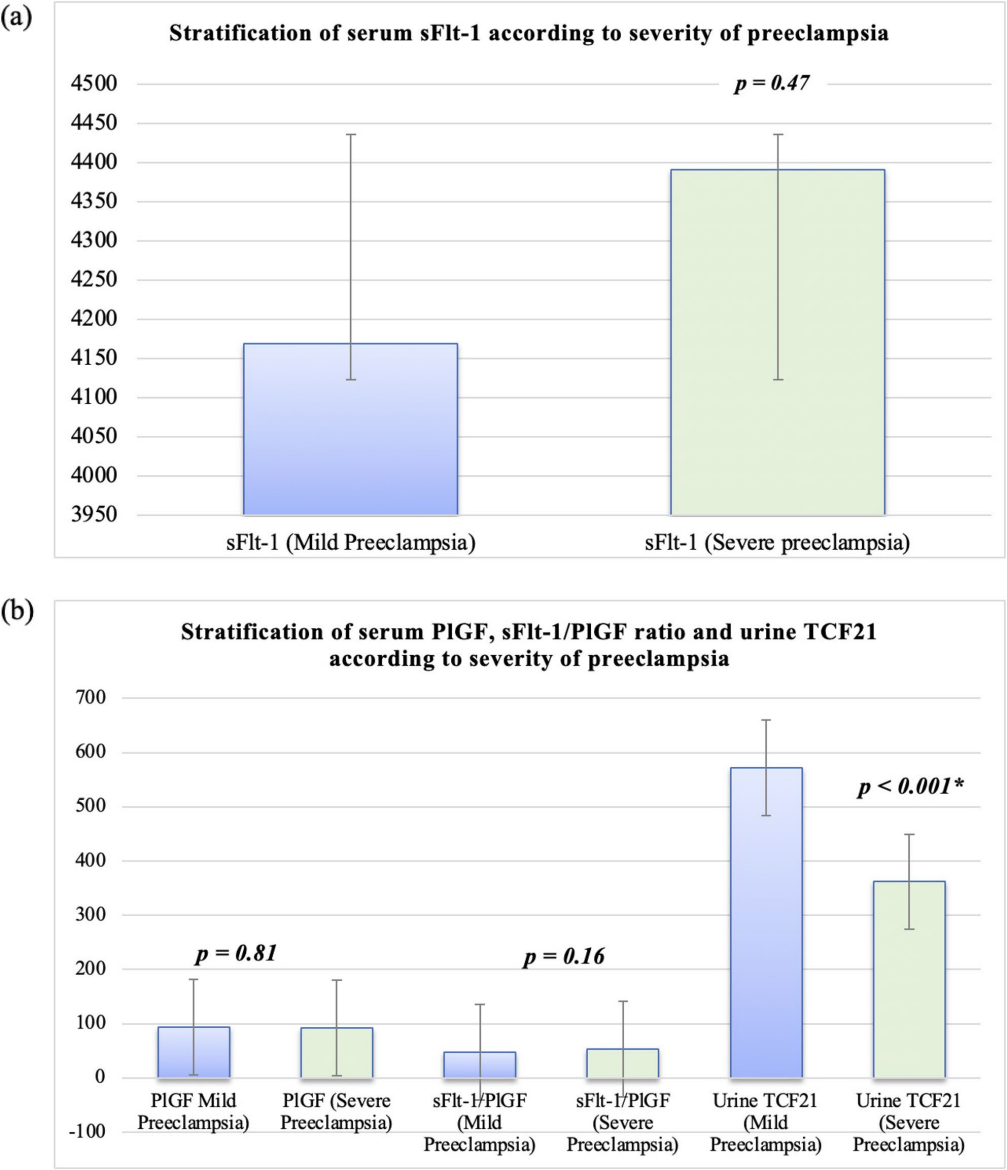
Stratification according to severity of preeclampsia. (**a**) Serum sFlt-1 by severity. (**b**) Serum PlGF, sFlt-1/PlGF ratio, and urinary TCF21 by severity

By severity, urinary TCF21 was significantly lower in severe vs. mild preeclampsia (361.9 ± 155.8 vs. 572.3 ± 300.9 pg/mL; *p* < 0.001) (Table 4; Fig. 4). The sFlt-1/PlGF ratio was numerically higher in severe cases but did not reach statistical significance (*p* = 0.16). Serum sFlt-1 and PlGF did not differ significantly between mild and severe groups (Table 4). Taken together, these findings suggest that while the angiogenic imbalance characterizes preeclampsia in general, urinary TCF21 may be more sensitive to gradations in disease severity than to timing of onset.

**Table 4:** Levels of angiogenic factors (serum sFlt-1, serum PlGF, sFlt-1/PlGF ratio), and urinary TCF21 in mild and severe preeclampsia

		Severity of preeclampsia		*p* value
		Mild *N* = 92	Severe *N* = 68	
Serum sFlt-1	Range	2455–5400	2035–5400	0.47
	Mean ± SD	4169 ± 833.5	4390.3 ± 758.3	
Serum PlGF	Range	50–250	55–270	0.81
	Mean ± SD	94.1 ± 36.9	92.1 ± 45	
sFlt-1/PlGF	Range	15.09–78.8	16.96–89.55	0.16
	Mean ± SD	47.93 ± 14.1	53.1 ± 16.2	
Urine TCF21	Range	125–1190	155–935	< 0.001*
	Mean ± SD	572.3 ± 300.9	361.9 ± 155.8	

Data are presented as mean ± SD (range). Comparisons between mild and severe preeclampsia groups were performed using the independent-samples t test

*sFlt-1* soluble fms-like tyrosine kinase-1, *PlGF* Placental growth factor, *TCF21* Transcription factor 21, sFlt-1/PlGF ratio is unitless

*Significant at *p* < 0.05

### Logistic regression and multivariable model performance

#### Urine TCF21 is associated with preeclampsia case status in univariate logistic regression

In univariate logistic regression, serum sFlt-1, serum PlGF (ridge-penalized model due to near-complete separation), and urinary TCF21 were each significantly associated with preeclampsia case status (Table 5). Higher sFlt-1 and urinary TCF21 was associated with higher odds of preeclampsia, whereas higher PlGF was associated with lower odds (Table 5).

**Table 5:** Univariate logistic regression analysis for the discrimination of preeclampsia:

	OR	95% CI	*p*-value
Serum sFlt-1 (per 100 pg/mL increase)	1.110	1.101–1.120	< 0.001***
Serum PlGF (per 10 pg/mL increase)^†^	0.243	0.063–0.938	0.040*
Urine TCF21 (per 50 pg/mL increase)	1.489	1.238–1.815	< 0.001***

Data are presented as odds ratios (ORs) with 95% confidence intervals (CIs) from univariate logistic regression models with preeclampsia as the outcome. Biomarkers were scaled for interpretability (sFlt-1 per 100 pg/mL, PlGF per 10 pg/mL, and urinary TCF21 per 50 pg/mL; values for a 100 pg/mL increase can be obtained by squaring the 50-unit OR)

*sFlt-1* soluble fms-like tyrosine kinase-1, *PlGF* Placental growth factor, *TCF21* Transcription factor 21

^†^Ridge-penalized logistic regression was used for PlGF because of near-complete separation between preeclampsia and control PlGF values

*p < 0.05; **p < 0.01; ***p < 0.001

### Multivariate model

In multivariable logistic regression adjusting for clinical covariates and angiogenic status, urinary TCF21 remained independently associated with preeclampsia (adjusted OR 2.83 per 50 pg/mL; 95% CI 1.49–5.59; *p* = 0.002) (Table 6). (For reference, this corresponds to an adjusted OR of approximately 7.99 per 100 pg/mL.) Independent contributions were also observed for the sFlt-1/PlGF ratio, gestational age, nulliparity, systolic blood pressure, and UPCR (Table 6). The full model showed good calibration (Hosmer–Lemeshow *p* = 0.502) and strong discrimination (AUC 0.87, 95% CI 0.82–0.91) (Tables 6 and 7). In stratified analyses, model discrimination remained high across severity and onset subgroups (Table 7).

**Table 6:** Multivariable logistic regression analysis for discrimination of preeclampsia

Variable	β-coefficient	Adjusted OR (95% CI)	*p*-value
TCF21 (per 50 pg/mL increase)	1.039	2.827 (1.489–5.585)	0.002**
sFlt-1/PlGF ratio	0.138	1.148 (1.075–1.226)	< 0.001***
Gestational age (weeks)	-0.117	0.890 (0.815–0.971)	0.008**
Nulliparity	0.615	1.850 (1.120–3.055)	0.016*
Systolic BP (per 10 mm Hg)	0.199	1.220 (1.078–1.380)	0.002**
UPCR (g/g)	0.833	2.301 (1.448–3.657)	< 0.001***

Data are presented as β-coefficients and adjusted odds ratios (ORs) with 95% confidence intervals (CIs) from a multivariable logistic regression model with preeclampsia as the outcome. Model Statistics: Nagelkerke R² = 0.412; -2 Log Likelihood = 172.4; Hosmer-Lemeshow Test: χ²=7.32, p = 0.502. TCF21 ORs correspond to a 50 pg/mL increase (values for a 100 pg/mL increase can be obtained by squaring the 50-unit OR); the sFlt-1/PlGF ratio is per 1-unit, systolic blood pressure (BP) per 10 mm Hg, and UPCR in g/g

*TCF21* Transcription factor 21, *sFlt-1/PlGF* soluble fms-like tyrosine kinase-1 to placental growth factor ratio, *UPCR* Urine protein–creatinine ratio

*p < 0.05; **p < 0.01; ***p < 0.001

**Table 7:** Stratified multivariable analysis according to severity and onset

Subgroup	TCF21 aOR per 50 pg/mL(95% CI)	*p*-value	AUC (95% CI)
All PE Cases (*n* = 160)	2.827 (1.489–5.585)	0.002**	0.87 (0.82–0.91)
Mild PE (*n* = 92)	2.440 (1.161–5.070)	0.018*	0.79 (0.72–0.86)
Severe PE (*n* = 68)	3.437 (1.645–7.457)	0.002**	0.85 (0.78–0.92)
Early-onset (*n* = 81)	3.273 (1.565–6.773)	0.002**	0.88 (0.82–0.94)
Late-onset (*n* = 79)	2.105 (1.051–4.176)	0.038*	0.81 (0.74–0.88)

Data are presented as adjusted odds ratios (aORs) with 95% confidence intervals (CIs) for urinary TCF21 from multivariable logistic regression models stratified by preeclampsia severity and onset, together with the corresponding area under the receiver operating characteristic curve (AUC) and 95% CI for each model. TCF21 aORs correspond to a 50 pg/mL increase (values for a 100 pg/mL increase can be obtained by squaring the 50-unit OR)

*PE* Preeclampsia, *TCF21* Transcription factor 21

*p < 0.05; **p < 0.01; ***p < 0.001

Sequential likelihood-ratio testing demonstrated improved model fit with stepwise addition of predictors: adding the sFlt-1/PlGF ratio to clinical factors improved fit, and adding TCF21 provided a further significant improvement (Table 8).

**Table 8:** Model comparisons based on -2 log-likelihood and likelihood ratio tests

Model	-2 LL	Δχ² (df)	*p*-value
Clinical factors only	215.3	-	-
+ sFlt-1/PlGF ratio	189.6	25.7 (1)	< 0.001*
+ TCF21	172.4	17.2 (1)	< 0.001*

−2LL, − 2 log-likelihood; Δχ², change in chi-square; df, degrees of freedom

The “Clinical factors only” model includes gestational age, nulliparity, systolic blood pressure, and proteinuria. The “+ sFlt/PlGF ratio” model additionally includes the sFlt-1/PlGF ratio as the angiogenic variable. The “+ TCF21” model further includes urinary TCF21. *p* values are derived from likelihood ratio tests comparing each model with the preceding, nested model

*p < 0.05 was considered statistically significant

### ROC-based diagnostic performance and threshold metrics

#### Urine TCF21 is a potential diagnostic test for preeclampsia

**Fig. 5:**
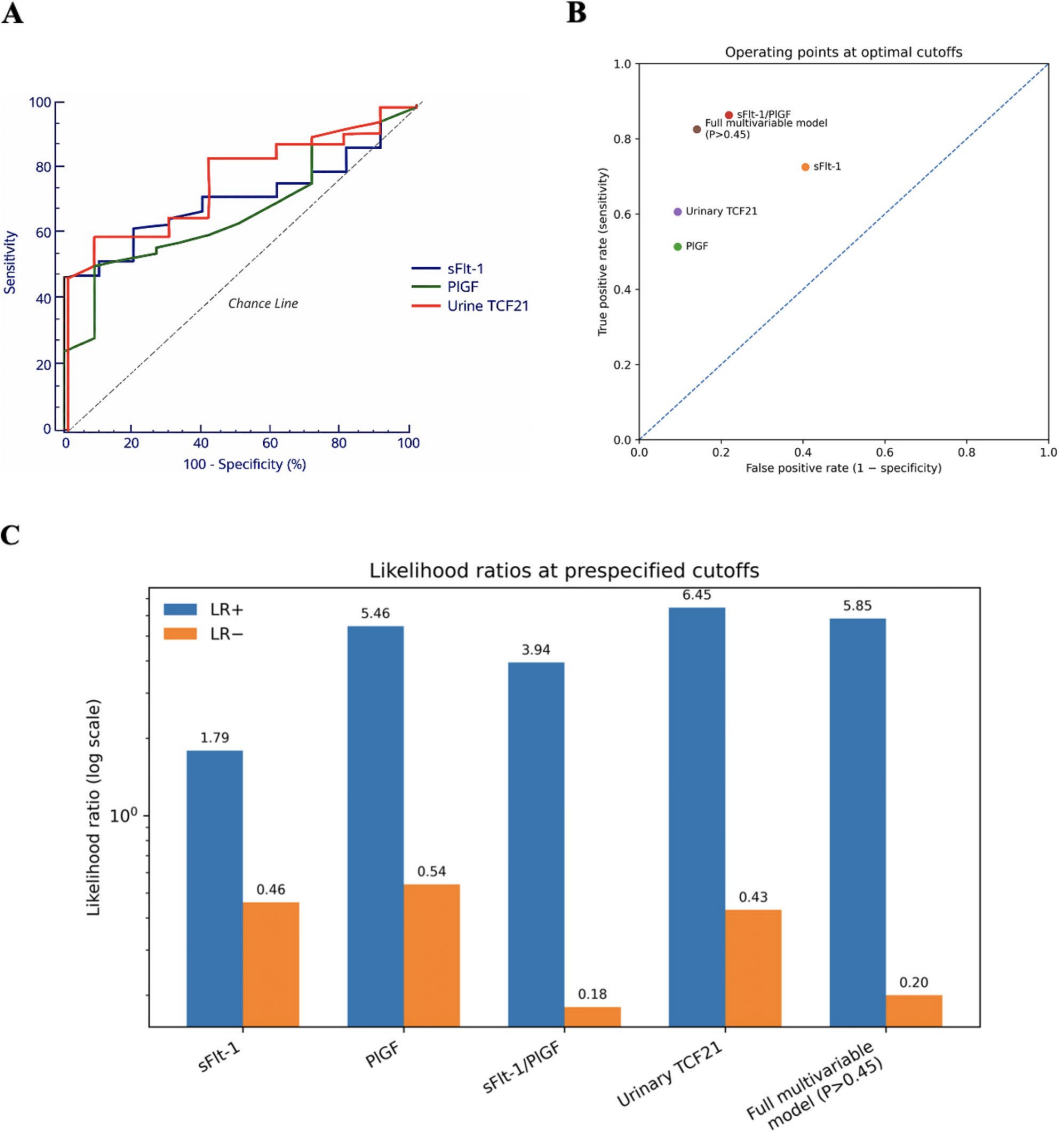
Diagnostic discrimination and threshold performance of urinary TCF21 and comparator biomarkers. (**A**) ROC curve for urinary TCF21 in discriminating preeclampsia from controls (AUC = 0.769). At the prespecified cutoff of > 370 pg/mL, sensitivity was 60.6% and specificity was 90.6% (*p* < 0.001). (**B**) Operating points at prespecified optimal cutoffs: scatter plot of sensitivity (true-positive rate) versus false-positive rate (1 − specificity) for each biomarker and for the multivariable model threshold. (**C**) Likelihood ratios at prespecified cutoffs: LR + and LR − are shown on a log scale for each biomarker and for the multivariable model threshold, summarizing rule-in (higher LR+) and rule-out (lower LR−) performance

ROC analysis comparing individual biomarkers showed that the sFlt-1/PlGF ratio had the highest single-marker discrimination (AUC 0.812), followed by urinary TCF21 (AUC 0.769) (Table 9; Fig. 5). At the prespecified optimal cutoff of > 370 pg/mL, urinary TCF21 achieved 60.6% sensitivity and 90.6% specificity, corresponding to LR + 6.45 and LR − 0.43 (Table 9). PPV/NPV were not reported because they depend on disease prevalence, which cannot be inferred from a case–control design.

**Table 9:** ROC analysis of serum sFlt-1, serum PlGF, sFlt-1/PlGF ratio, and urinary TCF21 for diagnosis of preeclampsia

	Serum sFlt-1 (pg/mL)	Serum PlGF (pg/mL)	sFlt-1/PlGF	Urine TCF21 (pg/mL)
Optimal cutoff	3875	84	85	370
AUC	0.715	0.678	0.812	0.769
95% CI	0.651–0.773	0.613–0.739	0.754–0.862	0.708–0.822
*p* value	< 0.001*	< 0.001*	< 0.001*	< 0.001*
Sensitivity	72.5%	51.3%	86.3%	60.6%
Specificity	59.4%	90.6%	78.1%	90.6%
LR+	1.79	5.46	3.94	6.45
LR-	0.46	0.54	0.18	0.43
Youden index (J)	0.319	0.419	0.644	0.512

Data are based on receiver operating characteristic (ROC) curve analysis for discrimination between preeclampsia cases and normotensive pregnant controls

Optimal cutoffs were chosen to maximize Youden’s index (sensitivity + specificity − 1). Predictive values (PPV/NPV) are not reported because they depend on disease prevalence, which cannot be estimated from a case-control design

*AUC* Area under the ROC curve, *CI* Confidence interval, *LR+* Positive likelihood ratio, *LR-* Negative likelihood ratio, *sFlt-1* soluble fms-like tyrosine kinase-1, *PlGF* Placental growth factor, *TCF21* Transcription factor 21. The sFlt-1/PlGF ratio is unitless

*p < 0.05 was considered statistically significant

In subgroup ROC analyses, biomarker discrimination remained clinically relevant. For early-onset preeclampsia, TCF21 had AUC 0.792 and sFlt-1/PlGF had AUC 0.851 (Table 10). For severe preeclampsia, TCF21 had AUC 0.735 and sFlt-1/PlGF had AUC 0.843 (Table 11).

**Table 10:** Diagnostic performance of biomarkers in early-onset preeclampsia (*n* = 81)

Biomarker	AUC	Sensitivity	Specificity
TCF21	0.792	65.4%	85.9%
sFlt-1	0.753	79.0%	65.6%
PlGF	0.715	58.0%	87.5%
sFlt-1/PlGF	0.851	90.1%	81.3%

Data are based on receiver operating characteristic (ROC) analysis for discrimination of early-onset preeclampsia (*n* = 81) from normotensive pregnant controls using the indicated biomarkers at their respective optimal cutoffs

*AUC* Area under the ROC curve, *TCF21* Transcription factor 21, *sFlt-1* soluble fms-like tyrosine kinase-1, *PlGF* Placental growth factor, *sFlt-1/PlGF* soluble fms-like tyrosine kinase-1 to placental growth factor ratio

**Table 11:** Diagnostic performance of biomarkers in severe preeclampsia (*n* = 68)

Biomarker	AUC	Sensitivity	Specificity
TCF21	0.735	55.9%	89.1%
sFlt-1	0.781	85.3%	62.5%
PlGF	0.702	63.2%	82.8%
sFlt-1/PlGF	0.843	91.2%	75.0%

Data are based on receiver operating characteristic (ROC) analysis for discrimination of severe preeclampsia (*n* = 68) from normotensive pregnant controls using the indicated biomarkers at their respective optimal cutoffs

*AUC* Area under the ROC curve, *TCF21* Transcription factor 21, *sFlt-1* soluble fms-like tyrosine kinase-1, *PlGF* Placental growth factor, *sFlt-1/PlGF* soluble fms-like tyrosine kinase-1 to placental growth factor ratio

### Model probability thresholds and clinical classification

**Fig. 6:**
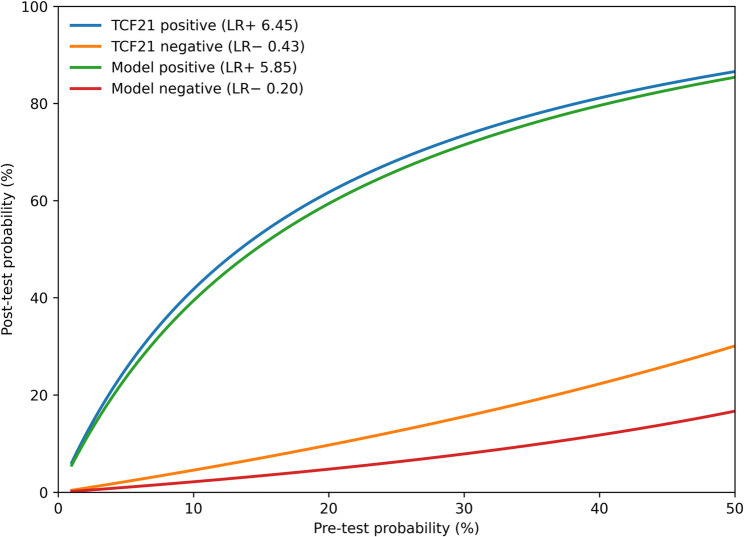
Pre- to post-test probability transformation using likelihood ratios. Curves show how a positive or negative test result transforms pre-test probability into post-test probability using LR + and LR − for urinary TCF21 and the multivariable model

Using the final multivariable model, classification performance varied across probability thresholds (Table 12; Fig. 6). A lower threshold (> 0.30) prioritized sensitivity (95.1%) with moderate specificity (62.5%) and LR − 0.08. An intermediate threshold (> 0.45) provided a more balanced trade-off (sensitivity 82.5%, specificity 85.9%; LR + 5.85, LR − 0.20). A higher threshold (> 0.65) prioritized specificity (93.8%) with LR + 11.02 but reduced sensitivity (68.3%) (Table 12).

**Table 12:** Clinical impact of probability thresholds for preeclampsia discrimination

Probability Threshold	Sensitivity (%)	Specificity (%)	LR+	LR-
> 0.30 (Rule-out)	95.1%	62.5%	2.54	0.08
> 0.45 (Optimal)	82.5%	85.9%	5.85	0.2
> 0.65 (Rule-in)	68.3%	93.8%	11.02	0.34

Data are derived from the final multivariable logistic regression model for preeclampsia, incorporating clinical variables, the sFlt-1/PlGF ratio, and urinary TCF21. Sensitivity, specificity, and likelihood ratios are shown for three illustrative discriminative-probability thresholds. LR + = sensitivity/(1 − specificity); LR- = (1 − sensitivity)/specificity. TCF21, transcription factor 21

## Discussion

In this case-control study, urinary TCF21 was higher in women with preeclampsia than in normotensive pregnant controls and remained independently associated with preeclampsia after adjustment for clinical and angiogenic variables. Urinary TCF21 demonstrated moderate single-marker discrimination (AUC 0.769) and was inferior to the sFlt-1/PlGF ratio, which provided the strongest single-marker performance. Nevertheless, adding urinary TCF21 to multivariable models improved discrimination and model fit, supporting the concept that a podocyte-related renal-axis signal may complement the placental angiogenic axis at the time of clinical evaluation. Given the diagnostic-time case-control design, these findings should be interpreted as exploratory and hypothesis-generating rather than establishing a validated clinical diagnostic classifier.

Preeclampsia is increasingly understood as a systemic syndrome initiated by placental dysfunction and propagated through angiogenic imbalance and maternal endothelial injury. Elevated sFlt-1 and reduced PlGF are central to this paradigm and have become established tools for risk stratification in clinical practice [1]. Early-onset disease (< 34 weeks’ gestation) is more strongly driven by placental insufficiency, whereas late-onset cases (≥34 weeks’ gestation) more often reflect maternal cardiovascular stress [12, 13]. Recent prospective clinical studies show that early-onset cases exhibit patterns of placental insufficiency, while late-onset cases are characterized by cardiac maladaptation, with more severe cardiac impairment observed in preterm compared with term preeclampsia [14, 15]. Consistent with this biology, we observed the expected anti-angiogenic profile in cases, and the sFlt-1/PlGF ratio demonstrated the strongest single-marker discrimination for preeclampsia in our cohort. However, angiogenic markers primarily reflect placental and systemic vascular stress, and they do not fully capture the downstream renal phenotype that contributes to morbidity and guides management decisions. Our results extend the current biomarker framework by linking the placental–vascular axis to podocyte biology through a measurable, urine-based marker.

TCF21 is a transcription factor expressed in podocytes and mesenchymal precursors and is essential for normal glomerular development and maintenance. Experimental work has demonstrated that loss or dysregulation of TCF21 disrupts podocyte differentiation and promotes glomerular scarring, whereas selective deletion of TCF21 in adult podocytes accelerates progressive renal injury. In clinical studies of glomerular disease, urinary or tissue TCF21 expression has been associated with podocyte injury and disease activity. In this context, our observation that urinary TCF21 is elevated in preeclampsia is biologically plausible, as podocyturia is a well-described feature of the syndrome and reflects ongoing glomerular injury. The elevation of urinary TCF21 is likely due to compensatory upregulation in the setting of glomerular and endothelial injury, mitigating apoptosis and preserving cytoskeletal integrity. Supporting evidence comes from experimental studies showing increased TCF21 expression in human and rat kidney tissue following podocyte injury, with parallel urinary excretion patterns [7]. In conditional knockout mouse models, deletion of TCF21 at the capillary-loop stage caused focal segmental glomerulosclerosis (40% incidence by 5 weeks), whereas earlier deletion universally induced renal failure, highlighting its central role in sustaining podocyte function [16].

An unexpected yet hypothesis-generating finding was the inverse association between TCF21 levels and preeclampsia severity. While mild preeclampsia showed higher urinary TCF21, levels were lower in severe disease. This pattern may reflect dynamic biomarker kinetics (including timing relative to symptom onset), podocyte stress/adaptive responses, altered podocyte shedding, or depletion in advanced disease; however, it should be interpreted cautiously given the cross-sectional design and potential influence of urine dilution and assay standardization.

Several non-exclusive mechanistic explanations may underlie this observation. First, early podocyte stress – driven by endothelial dysfunction, hypertension, and angiogenic imbalance – may trigger compensatory upregulation of TCF21 as part of a protective transcriptional program. TCF21 is known to promote podocyte survival, maintain cytoskeletal integrity, and mitigate apoptosis in response to injury [7]. Increased urinary TCF21 in mild disease could thus reflect active podocyte adaptation and enhanced shedding of TCF21-positive podocytes or podocyte-derived vesicles.

In severe or advanced preeclampsia, however, sustained injury may lead to podocyte depletion through detachment, apoptosis, or ferroptosis, reducing the cellular reservoir capable of mounting a compensatory TCF21 response [16]. This “exhaustion” of podocyte resilience could result in diminished TCF21 expression and urinary excretion, despite greater glomerular damage. This hypothesis aligns with experimental models of progressive glomerular disease, where TCF21 expression declines as structural injury accumulates and podocyte density falls [7, 16].

Alternatively, altered transcriptional regulation in severe preeclampsia – possibly mediated by hypoxia, oxidative stress, or inflammatory cytokines – could suppress TCF21 expression independently of podocyte loss. Furthermore, severe preeclampsia is often accompanied by impaired renal filtration and tubular function, which might affect the urinary clearance or degradation of TCF21, though this remains speculative.

Importantly, this inverse severity relationship contrasts with traditional angiogenic markers (e.g., sFlt-1/PlGF ratio), which typically rise with disease progression [11, 17]. This divergence reinforces the concept that TCF21 captures a distinct renal podocyte phenotype, potentially reflecting podocyte reserve or adaptive capacity rather than mere injury magnitude. Future studies measuring podocyte count (e.g., podocyturia), renal biopsy TCF21 expression, and longitudinal urinary TCF21 trajectories will be essential to dissect these mechanisms and determine whether declining TCF21 signals irreversible glomerular damage. Interpretation is limited by diagnostic-time sampling and non-normalized urinary concentrations and should be confirmed in prospective studies with serial sampling and creatinine-normalized reporting.

Our findings regarding traditional risk factors also align with prior literature. The higher random blood glucose observed among preeclamptic patients reinforces the bidirectional relationship between preeclampsia and disordered glucose metabolism. Pre-existing diabetes (type 1, type 2 or gestational) is a well-established risk factor for preeclampsia, while a history of preeclampsia increases the long-term risk of diabetes in later life, likely reflecting a shared substrate of insulin resistance and endothelial dysfunction [18]. The convergence of angiogenic imbalance, podocyte stress (as indexed by TCF21) and metabolic dysregulation in our cohort underscores the multifactorial nature of preeclampsia and its implications for maternal cardiovascular and renal health beyond pregnancy.

From a diagnostic perspective, our data support the continued use of the sFlt-1/PlGF ratio, as a strong tool for ruling in or ruling out preeclampsia, in line with clinical studies and contemporary guideline recommendations [2, 11, 17, 19–22]. In our study, this ratio provided the highest overall discrimination among single biomarkers. Compared with angiogenic markers, TCF21 captured a different dimension of preeclampsia. While sFlt-1/PlGF clearly distinguished preeclampsia from normotensive pregnancy, its ability to separate mild from severe disease in our cohort was limited. In contrast, urinary TCF21 showed a closer relationship with clinical severity and with markers of renal involvement such as proteinuria and platelet count. These differences suggest that angiogenic markers primarily reflect placental and systemic endothelial stress, whereas TCF21 may capture a distinct podocyte-related renal dimension rather than directly quantifying overall renal injury severity. Prior studies have evaluated podocyte markers such as nephrin, podocalyxin, and podocyte-specific mRNA in preeclampsia, but to our knowledge, urinary TCF21 has not been systematically assessed in this setting. Our results therefore add to the growing body of evidence that podocyte-derived markers can complement angiogenic factors for more comprehensive characterization of preeclampsia.

**Fig. 7:**
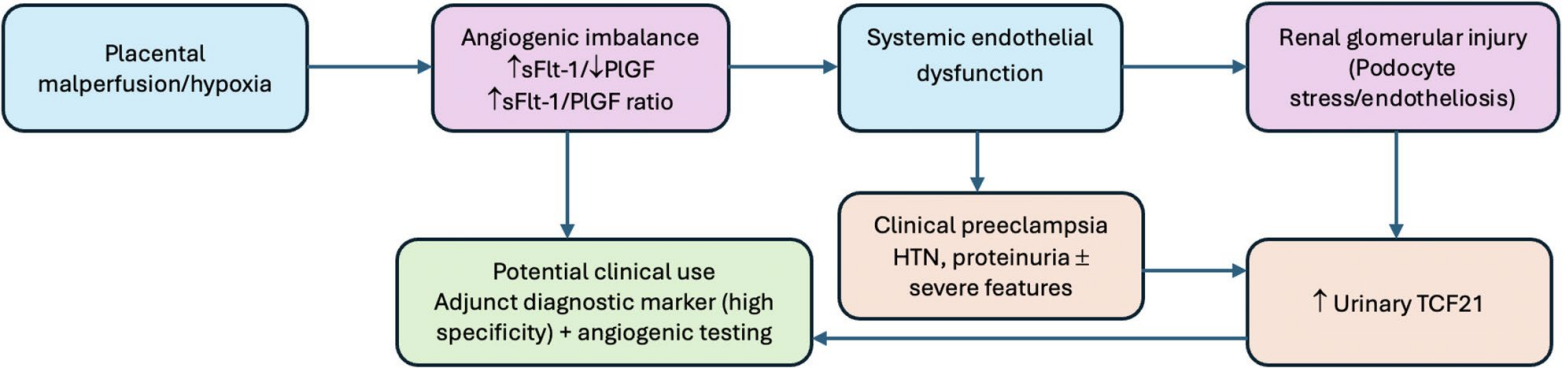
Proposed translational framework linking angiogenic imbalance and urinary TCF21. Conceptual, hypothesis-generating schematic summarizing a biologically plausible (non-causal) pathway in which placental malperfusion and angiogenic imbalance (↑sFlt-1, ↓PlGF) may contribute to endothelial and renal injury with downstream podocyte stress, potentially accompanied by increased urinary TCF21. This framework is intended to guide future mechanistic and longitudinal studies and should not be interpreted as evidence of causality

The conceptual framework in Fig. 7 integrates these observations into a translational pathway: placental malperfusion triggers a systemic angiogenic imbalance characterized by increased anti-angiogenic signaling (↑sFlt-1) and reduced pro-angiogenic support (↓PlGF). This imbalance can promote widespread endothelial dysfunction and microvascular injury, with the kidney representing a major target organ in preeclampsia. Renal endothelial injury and downstream glomerular/tubulointerstitial stress may contribute to cellular activation and transcriptional responses that culminate in increased urinary shedding of TCF21. Within this conceptual model, urinary TCF21 functions as a noninvasive readout of renal involvement occurring along the angiogenic–endothelial injury axis, potentially complementing established angiogenic biomarkers (including the sFlt-1/PlGF ratio). Clinically, this supports the use of urinary TCF21 as an adjunct diagnostic marker—particularly in settings where symptomatology is nonspecific or where a rapid, urine-based test could aid triage. Importantly, Fig. 7 is presented as a biologically plausible pathway that should be corroborated by mechanistic and longitudinal studies linking dynamic changes in angiogenic markers, renal injury phenotypes, and urinary TCF21 trajectories.

Our probability-threshold analyses further highlight how combined biomarker models may support bedside decision-making. Lower model thresholds prioritize sensitivity and may be useful for triage and minimizing missed cases, whereas higher thresholds prioritize specificity and may support confirmatory decisions such as intensified surveillance, hospitalization, corticosteroid administration for fetal lung maturation, or delivery planning. Within such a strategy, urinary TCF21 may be most helpful in refining risk in patients with intermediate angiogenic results or ambiguous clinical presentations—particularly in resource-limited settings where rapid, urine-based testing may be easier to implement than repeat angiogenic assays.

This study has several strengths. We enrolled a well-characterized cohort of women with rigorously defined preeclampsia and matched normotensive controls; collected detailed clinical and laboratory data; and measured angiogenic and podocyte-related biomarkers in parallel using standardized assays. We also went beyond simple group comparisons by incorporating TCF21 into multivariable models and exploring clinically meaningful probability thresholds, which moves the analysis closer to real-world decision-making. Finally, by examining both early- and late-onset as well as mild and severe preeclampsia, we were able to explore how TCF21 behaves across different clinical phenotypes.

However, several limitations should be acknowledged. First, this was a single-center study with a relatively modest sample size—particularly within certain clinical subgroups—which may reduce the precision of effect estimates and limit the ability to detect subtle differences between phenotypes. Second, the case–control design with biomarker measurement at the time of clinical evaluation/diagnosis supports diagnostic discrimination at presentation but does not permit assessment of temporal trajectories of urinary TCF21 or its ability to identify preeclampsia before clinical onset; therefore, our findings should not be interpreted as evidence for early- or mid-pregnancy screening. Future prospective longitudinal cohorts with serial sampling across gestation are needed to evaluate predictive utility and clinical actionability. Third, because participants were recruited from a single geographic and healthcare setting, generalizability to other populations with different baseline risk profiles, ethnic backgrounds, or antenatal-care patterns remains uncertain. Fourth, urinary TCF21 assays are not yet standardized for routine clinical use, and we quantified TCF21 as an absolute urinary concentration, which may be influenced by urine dilution; moreover, urinary TCF21 showed only moderate discrimination and was inferior to the sFlt-1/PlGF ratio, thus, the proposed cutoffs and probability thresholds should be considered exploratory until validated in independent external cohorts and, ideally, with creatinine-normalized reporting and inter-laboratory reproducibility. Fifth, model performance may be overestimated because development and evaluation were performed in the same cohort and because some variables included in multivariable models (e.g., blood pressure and proteinuria metrics) are closely related to diagnostic definition; therefore, external validation and sensitivity analyses excluding diagnostic-defining components are warranted. Finally, we did not assess long-term maternal renal or cardiovascular outcomes; thus, the prognostic implications of urinary TCF21 beyond pregnancy remain unknown. The observed inverse association between urinary TCF21 and disease severity should be interpreted cautiously and considered hypothesis-generating, requiring mechanistic and longitudinal studies to clarify whether it reflects biological dynamics rather than a simple monotonic relationship with injury burden.

In summary, urinary TCF21 is a novel, non-invasive biomarker associated with preeclampsia and provides incremental discriminative value beyond angiogenic markers and clinical variables. Its inverse association with severity suggests it may index a dynamic podocyte response or reserve rather than simply mirroring injury burden. Prospective, longitudinal studies in diverse populations are warranted to validate these findings, establish clinically robust thresholds, and determine whether integrating urinary TCF21 into multi-marker algorithms improves maternal and perinatal outcomes.

## Conclusion

In this case-control study, urinary TCF21 emerged as a novel, non-invasive biomarker associated with preeclampsia and provided incremental discriminative value beyond clinical variables and the sFlt-1/PlGF ratio. Consistent with established literature, the sFlt-1/PlGF ratio showed the strongest single-marker discrimination, whereas urinary TCF21 demonstrated moderate performance and appears best interpreted as a complementary renal-axis marker rather than a replacement for angiogenic testing. It should not be interpreted as a stand-alone validated clinical diagnostic test. At the prespecified cutoff (> 370 pg/mL), urinary TCF21 showed high specificity in this cohort, suggesting potential adjunct confirmatory performance at presentation when combined with clinical and angiogenic assessment. The inverse association with disease severity is hypothesis-generating and should not be interpreted causally. Because biomarker measurements were obtained at diagnosis and the study used a case-control design without internal validation, cutoffs and probability thresholds should be regarded as exploratory and may be optimistically biased. Prospective longitudinal studies with independent external validation (including creatinine-normalized reporting and assay standardization) are needed to confirm clinical utility and to determine whether integrating urinary TCF21 into biomarker algorithms improves maternal and perinatal outcomes.

### Proposed clinical algorithm

**Fig. 8:**
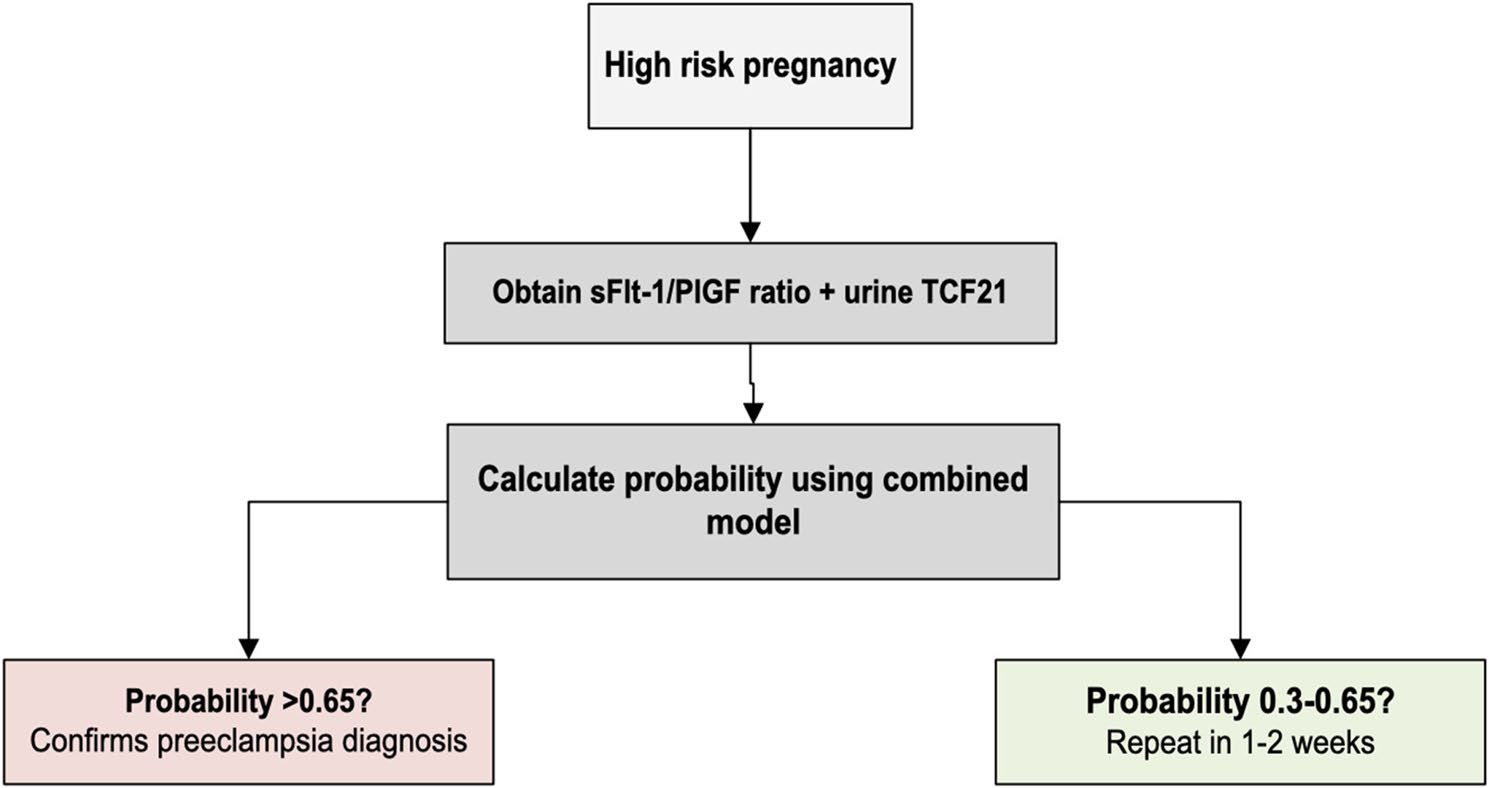
Proposed clinical algorithm for assessment of suspected preeclampsia in high-risk pregnancies using combined sFlt-1/PlGF ratio and urinary TCF21. This figure presents a hypothesis-generating stepwise approach informed by the present cohort and intended for evaluation in prospective settings. In women with suspected preeclampsia or high-risk features, initial assessment includes clinical evaluation (blood pressure, symptoms, and end-organ features) together with angiogenic testing using the sFlt-1/PlGF ratio as the primary placental-axis marker. A higher ratio (e.g., > 85 in this cohort) indicates increased likelihood of preeclampsia and warrants intensified clinical assessment and monitoring. Urinary TCF21 is then applied as an adjunct renal-axis marker: TCF21 > 370 pg/mL increased specificity in this dataset and may support an adjunct confirmatory interpretation when clinical findings are equivocal or when angiogenic values fall in an intermediate range. Where available, a combined multivariable model incorporating clinical variables, sFlt-1/PlGF, and urinary TCF21 can be used to generate an individualized probability estimate; higher thresholds (e.g., > 0.65) prioritize specificity for confirmatory triage, while lower thresholds prioritize sensitivity for minimizing missed cases (see Table 12; Fig. 6). All thresholds shown are derived from this single-center case-control dataset, are exploratory, and require external validation and assay standardization before any routine clinical use

Figure 8 presents a hypothesis-generating clinical algorithm for evaluating suspected preeclampsia in high-risk pregnancies by integrating placental and renal biomarkers. It proposes a stepwise approach: initial clinical assessment and angiogenic testing with the sFlt-1/PlGF ratio, followed by adjunct measurement of urinary TCF21 to increase specificity and refine risk in equivocal or intermediate presentations. The intent is to illustrate how complementary pathways could be integrated at presentation; it is not a validated diagnostic pathway, and all thresholds are exploratory and require prospective external validation before clinical implementation

## Data Availability

The datasets generated and/or analyzed during the current study are available from the corresponding author on reasonable request.

## Abbreviations

AUC: Area under the curve
BP: Blood pressure
CI: Confidence interval
ELISA: Enzyme–linked immunosorbent assay
HELLP: Hemolysis, elevated liver enzymes, low platelets
HO: 1–heme oxygenase–1
HRP: Horseradish peroxidase
IQR: Interquartile range
INR: International normalized ratio
OR: Odds ratio
aOR: adjusted odds ratio
PlGF: Placental growth factor
RBG: Random blood glucose
ROC: Receiver operating characteristic
sEng: soluble endoglin
sFlt: 1–soluble fms–like tyrosine kinase–1
SD: Standard deviation
TCF21: Transcription factor 21
UPCR: Urine protein–creatinine ratio
VEGF: Vascular endothelial growth factor
